# Beaming and enhanced transmission through a subwavelength aperture via epsilon-near-zero media

**DOI:** 10.1038/s41598-017-04680-y

**Published:** 2017-07-06

**Authors:** Hodjat Hajian, Ekmel Ozbay, Humeyra Caglayan

**Affiliations:** 1 Nanotechnology Research Center, Bilkent University, 06800 Ankara, Turkey; 2 Department of Physics, Bilkent University, 06800 Ankara, Turkey; 3 Department of Electrical and Electronics Engineering, Bilkent University, 06800 Ankara, Turkey; 4 UNAM-Institute of Materials Science and Nanotechnology, Bilkent University, 06800 Ankara, Turkey

## Abstract

We numerically validate and experimentally realize considerable funneling of electromagnetic energy through a subwavelength aperture that is covered with an epsilon-near-zero metamaterial (*ENZ*). The epsilon-near-zero metamaterial is composed of two layers of metasurfaces and operates at microwave frequencies. We demonstrate that the presence of the metamaterial at the inner and outer sides of the aperture not only lead to a significant enhancement in light transmission, but also cause a directional emission of light extracting from this hybrid system. In addition to these experimental results, we theoretically demonstrate the same concept in mid-IR region for a subwavelength gold aperture with indium tin oxide as an epsilon-near-zero material. Moreover, we found that using a dielectric spacer in-between the sunwavelength aperture and the *ENZ* medium, it is possible to red-shift the enhancement/directional frequency of the system.

## Introduction

Metamaterials^1^ have attracted significant attention over the past years due to their extraordinary optical properties. It is possible to manipulate the permittivity (*ε*), permeability (*μ*), and refractive index (*n*) of these structures to generate unusual values in a wide range of frequency. Double-negative metamaterials are the most well-known family of these systems with simultaneously negative values of *ε* and *μ*, and consequently negative values of the refractive index in microwave^2–4^, infrared^5–7^, and visible^8^ regions. More recently, extending from microwave to visible frequencies, *ε*-near-zero (*ENZ*)^9–14^, *μ*-near-zero (*MNZ*)^15^, and impedance matched index-near-zero^16–22^ structures have also been theoretically predicted and experimentally verified as another class of metamaterials called zero-index metamaterials (*ZIMs*). In *ZIMs*, due to the near-zero refractive index, the wavelength is relatively long and consequently light can pass with the characteristic of no-change in the spatial phase^16^. This characteristic has led to outstanding capabilities in molding the propagation of light. Squeezing the electromagnetic energy within very narrow channels^10^, designing matched zero-index systems^16^, shaping the radiation pattern of a source^9, 11, 12, 16, 22^, super-reflection and cloaking^17^, enhancing light transmission through a subwavelength aperture^12, 13^, and super-coupling^15^ can all be referred to as the highlighted application capabilities of these systems. Moreover, it has only been theoretically investigated that the presence of *ZIMs* at both sides of a subwavelength waveguide can lead to an increase in light transmission^23^. In case the Fabry-Perot resonance condition is satisfied in this system, due to coupling between zero-index modes and the Fabry-Perot resonances, considerable enhancement in the waveguide transmission can be observed. Similarly, it has been experimentally proven that the presence of an all-dielectric zero-index photonic crystal at the inner and outer sides of a photonic crystal waveguide can not only lead to a considerable enhancement in transmission, but it can also beam the extracted light from the hybrid structure^24^.

Furthermore, it has been proven that, by using a multilayered metal-dielectric fishnet metamaterial^25^ or a structure composed of multiple layers of dissimilar metasurfaces^26^, it is possible to achieve low-loss *ZIMs* in a broad-band region.

On the other hand, the presence of an *ENZ* material with Drude-like dispersion at the inner side of a subwavelength width metallic aperture can enhance the light transmission through it^12, 13^. It is noteworthy that transmission through a subwavelength aperture depends on the aperture diameter/width and the wavelength of the light. As defined in the standard diffraction theory by Bethe in 1944^27^, a circular aperture with a subwavelength diameter transmits electromagnetic waves rather poorly, and the exiting waves are fully diffracted in all directions. Enhanced transmission and beaming through a subwavelength aperture have been studied by several groups using corrugated metallic surfaces, see e.g. refs 28–31.

In the present paper, for the transverse-magnetic (TM) polarization of light, we experimentally investigate that using an *ENZ* metamaterial -instead of corrugated surfaces- at the inner side of a subwavelength metallic aperture, it is possible to enhance light transmission through the aperture. Moreover, we experimentally showed that by adding the *ENZ* metamaterial on both sides of the aperture, it is possible to obtain beaming, as well as enhance light transmission. Additionally, to further analyze these results, we use realistic (lossy) and low-loss ITO materials as homogeneous ENZ media. We numerically prove that by appropriately decreasing the losses of the ITO material, it is possible to simultaneously achieve enhanced transmission and beaming with the ENZ/A/ENZ system. Furthermore, by appropriately considering a dielectric spacer in-between the aperture and the metamaterial, the enhancement/directional frequency of the system can be tuned (red-shifted). To the best of our knowledge, the results presented in this paper have not been reported elsewhere.

## Results and Discussion

In this section, we first focus on the investigation of enhanced microwave transmission and beaming through a subwavelength aperture using a fishnet metamaterial that acts as *ZIM* around 12 *GHz*. Then, using Indium Thin Oxide (ITO), which can act as an *ENZ* material around 1.5 *μm* ^32^, we prove the enhanced transmission and beaming concept in the near-IR region.

### Funneling and beaming of electromagnetic waves with an epsilon-near-zero metamaterial

**Figure 1:**
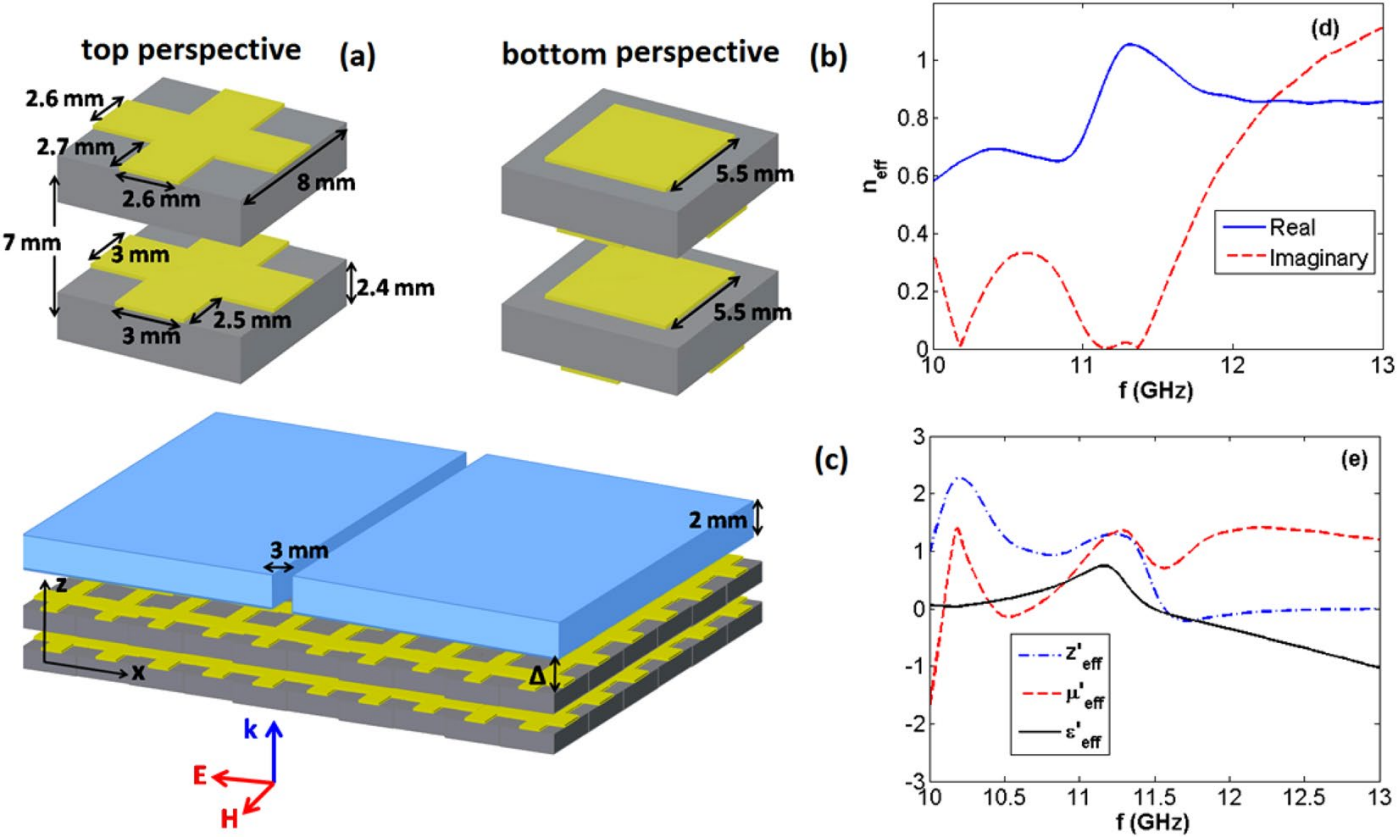
(**a**) and (**b**) show, respectively, top and bottom perspectives of each unit cell of the considered metamaterial with dissimilar metasurfaces. Regions in gray and yellow represent Teflon and copper layers, respectively. Panel (c) illustrates the *ZIM*/*A* case for which the metamaterial is placed at the inner side of the aperture, being separated by an air gap of width Δ. Notice that for the *ZIM*/*A*/*ZIM* structure, the aperture is symmetrically covered with the metamaterial. For the results presented here, we consider Δ ∼ 2 *mm*. Panels (d) and (e) represent the effective parameters of the metamaterial under our consideration calculated using the retrieval method. The solid-blue and dashed-red curves in panel (d) show the real and imaginary parts of the effective refractive index, respectively. Moreover, in panel (e) only the real parts of effective permittivity, permeability, and impedance are illustrated.

We schematically illustrate top and bottom perspectives of the unit cell of the metamaterial in panels (a) and (b) of Fig. 1. As shown in this figure, considering 24 *mm* (*f* = 12 *GHz*) as the operating wavelength, each unit cell is composed of two dissimilar metasurfaces with subwavelength inclusions. For the metamaterial, the copper elements are printed on either side of Teflon (*ε* = 2.2) boards. Panel (c) of Fig. 1 shows the system under our consideration for which the zero-index metamaterial is placed at the inner side of a subwavelength aperture (A) with an air gap of width Δ, referred to as a *ZIM*/*A* structure here. Moreover, *ZIM*/*A*/*ZIM* refers to the system for which the aperture is symmetrically covered by the metamaterial. Considering the normal plane wave incident on the structure, we extract the effective parameters of the metamaterial using the well-known retrieval method^25, 26, 33, 34^, as illustrated in panels (e) and (f) of Fig. 1.

**Figure 2:**
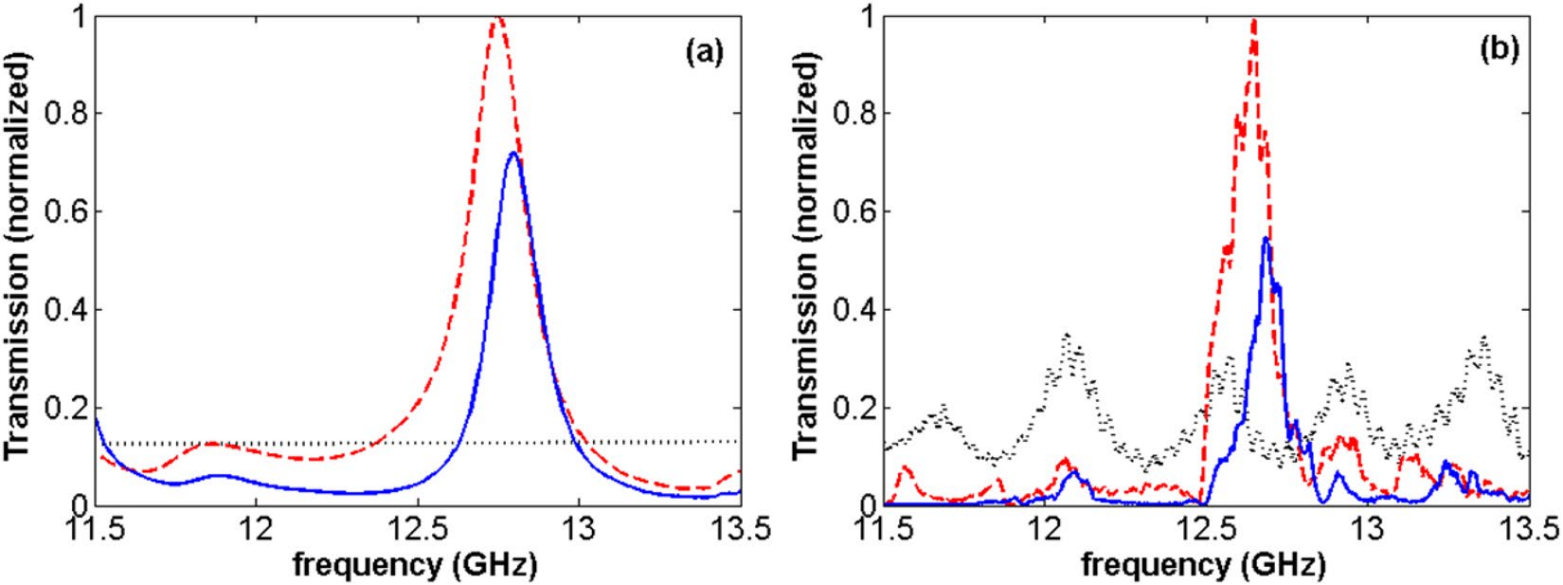
Numerical transmissions calculated for a bare aperture (dotted-black), ZIM/A structure (dashed-red), and *ZIM*/*A*/*ZIM* system (solid-blue) for Δ ∼ 2 *mm*. The experimental results are represented in panel (b), correspondingly.

Panels (d) and (e) of Fig. 1 show that the fishnet metamaterial acts as an index-near zero system in 10 to 11.8 *GHz* region, and it has *ENZ* characteristic within 9 to 13 *GHz* (the plots are illustrated in 10–13 *GHz*). To be able to validate the funneling characteristic of the metamaterial, using the finite-difference time-domain method (FDTD)^35^, we calculate light transmission through the subwavelength aperture in the presence of the metamaterial in Fig. 2(a).

As presented in Fig. 2(a), the presence of the metamaterial at the inner side of the aperture leads to a considerable enhancement in light transmission at *f* = 12.7 *GHz*. As shown by a dashed-red curve in Fig. S1(b) of the Supporting Information, a nearly seven fold enhancement in transmission is experimentally achieved in this case. In addition, it is seen that in the case for which the aperture is covered by the metamaterial, i.e. the *ZIM*/*A*/*ZIM* structure, light can funnel through the system at a three fold enhancement compared to the transmission of a bare aperture [check the enhancement value of the transmitted light for the *ZIM*/*A*/*ZIM* system at 12.7*GHz*, represented by a solid-blue curve in Fig. S1(b)]. As expected from the results presented in refs 13 and 23, since the metamaterial under our consideration has some losses, its presence at both sides of the aperture can decrease transmission compared to the case wherein we have it at the inner side only. At *f* = 12.7 *GHz*, ${n}_{eff}^{^{\prime} }=0.86$, ${z}_{eff}^{^{\prime} }$ = −0.01, ${\varepsilon }_{eff}^{^{\prime} }$ = −0.82, and ${\mu }_{eff}^{^{\prime} }$ = 1.29 and, consequently, the *ZIM* effectively acts like an *ENZ* medium. In a fair agreement with the numerical results, the experimental results, as illustrated in panel (b) of Fig. 2, demonstrate that the presence of *ZIM* at the inner side of the aperture considerably facilitates the funneling of light through it. Moreover, as shown by the solid-blue curve in Fig. 2(b), it is possible to keep the enhanced transmission high enough while we cover the aperture with the metamaterial on both sides.

**Figure 3:**
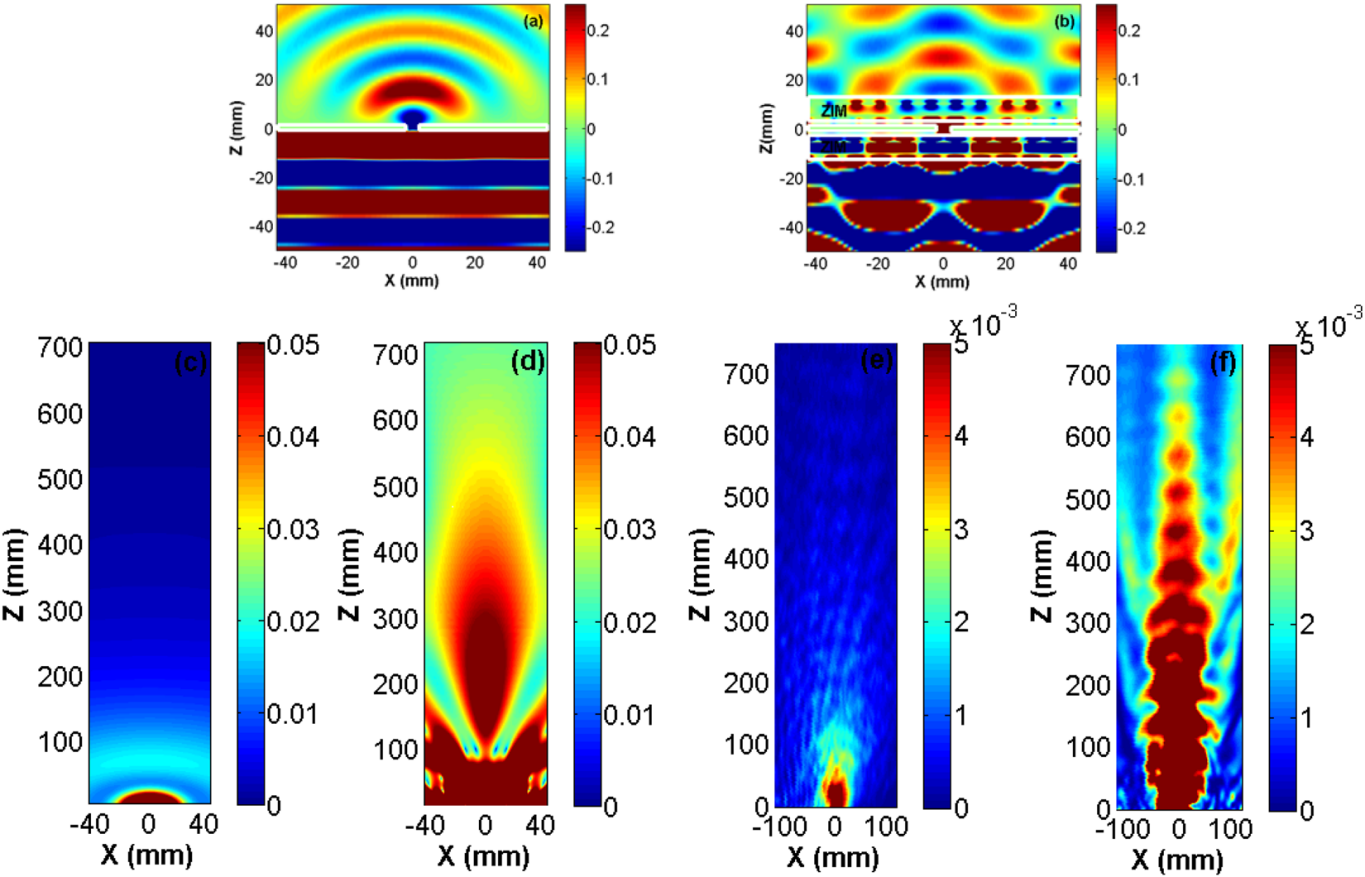
(**a**) and (**b**), respectively, illustrate numerical real part of the *E*_*x*_ component of the field distributions for the bare aperture and *ZIM*/*A*/*ZIM* system, at *f* = 12.7 *GHz*. In these figures, as indicated by the white regions, the aperture is placed at the origin, and *ZIM* is extended within *Z* = −12 to −1 *mm* (1 to 12 *mm*) at the inner (outer) side of it in panel (b). Panels (c) and (d) also numerically represent the corresponding far-field distributions of electric field intensity at the enhancement frequency. In agreement with the numerical results, the experimental far-filed mode profiles are illustrated in panels (e) and (f).

On the other hand, *ZIM* can enhance the directivity of the emission of a light source that leads to the strong beaming of the extracted electromagnetic wave^9, 11, 12, 16, 22^. To obtain further insight into the funneling mechanism of light and investigate the beaming effect, numerical near- and far-field electric mode profiles at the enhancement frequency, for the bare aperture and *ZIM*/*A*/*ZIM* structure, are illustrated in panels (a)–(d) of Fig. 3. The corresponding experimental far-field mode profiles are also represented in panels (e) and (f) of this figure.

It should be noted that for the results presented here, the aperture is placed at the origin. As is clearly shown in Fig. 3(a), without the presence of the metamaterial, light is considerably diffracted from it. The presence of *ZIM* at the inner side of the aperture significantly increases the coupling between free space and the aperture that is a consequence of the amplification of the amplitude of the electromagnetic field inside the *ZIM* medium^13^. The difference between the mode profiles in panels (a) and (b) of Fig. 3 in free space for *Z* < 0 is explanatory. Consequently, the increase in the coupling leads to the funneling of light through the aperture. Another remarkable point should be highlighted, as an explanation of Fig. 3(b), is the travel of light through *ZIM*/*A*/*ZIM* structure (extended from *Z* = −12 to 12 *mm*) with a quite negligible change in the phase. This is a direct consequence of the effective near-zero-index optical response of the metamaterial at the enhancement frequency; at this frequency, *Re*(*k*) = 0.86*k*_0_, where *k* and *k*_0_ are the wavenumber inside the index-near-zero medium and vacuum, respectively. It should be noted here that, as is discussed in the Supporting Information, by increasing the value of Δ, it is also possible to tune the resonance frequency from the upper edge of the *ENZ* region (*n*_*eff*_ ∼ 1) to smaller values, and thereby the above-mentioned enhancement effect can also be observed in the index-near-zero region in which the real values of *n*_*eff*_ is considerably small. Moreover, having the *ZIM* at the outer side of the aperture can also strongly couple the extracted light from the aperture to the free space. This mechanism leads to a collimation instead of diffraction; notice at the near-filed mode profile in Fig. 3(b) for *Z* > 12 *mm* and compare it to the one presented in panel (a) of Fig. 3. A drastic beaming, thus, is observed when we look at the far-filed distribution of the electric field intensity of the extracted light from the *ZIM*/*A*/*ZIM* system; see Fig. 3(d) and compare it with panel (c) of Fig. 3. In support of this point, we provide experimental results of the far-field measurement of the electric field intensity of light extracted from a bare aperture and the one covered on both sides with *ZIM* in Fig. 3(e) and (f), respectively. As clearly observed by the comparison of panels (e) and (f) with panels (c) and (d) of Fig. 3, the experimental results approve that the presence of *ZIM* at the outer side of the aperture leads to the beaming of the extracted light.

**Figure 4:**
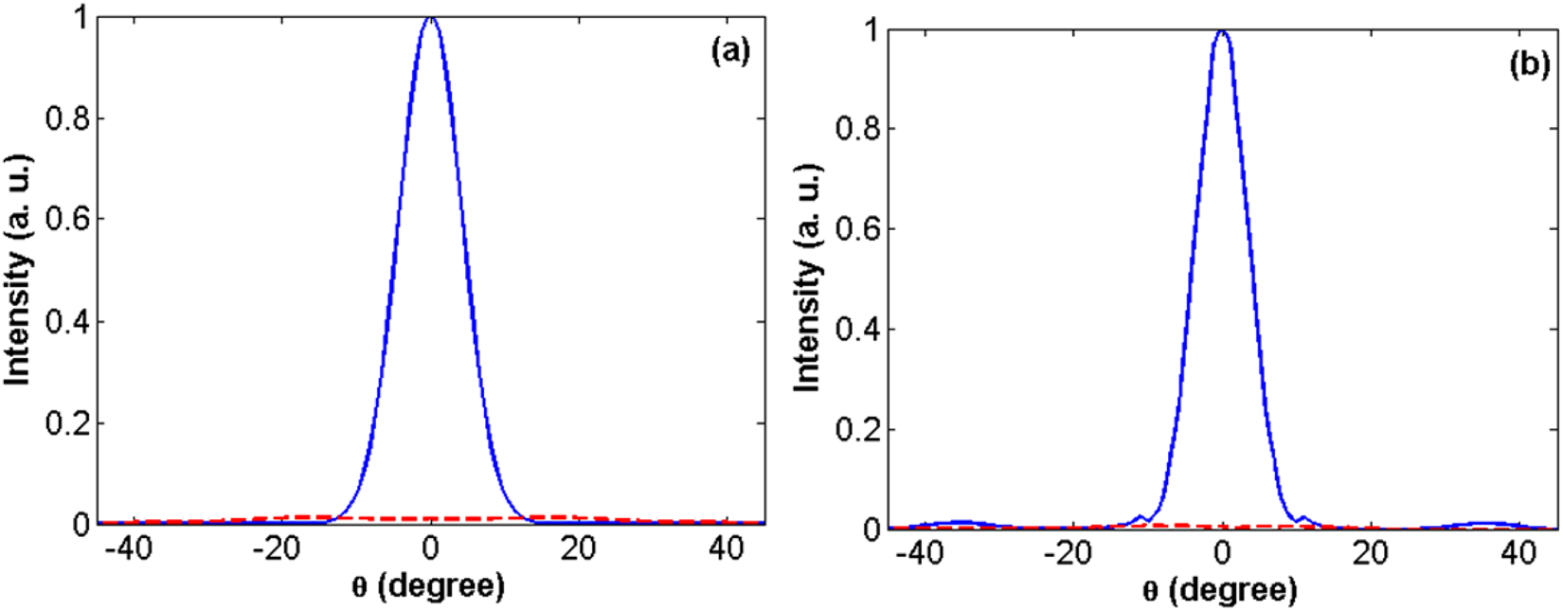
Dashed-red and solid-blue curves in panel (a) and (b), respectively, represents the calculated and measured far-field angular radiation patterns of the bare aperture and *ZIM*/*A*/*ZIM* hybrid structure, at *f* = 12.75 *GHz*.

In agreement with the results concluded from Fig. 3, and to illustratively examine the directionality of the hybrid *ZIM*/*A*/*ZIM* structure, the numerical far-field angular distribution of this system and a bare aperture, at *f* = 12.7 *GHz*, are presented in solid-blue and dashed-red curves in Fig. 4(a), respectively. As it is shown in this figure, the beaming behavior represented in Fig. 3 appears as an enhanced directional emission. This fact is also experimentally verified in panel (b) of Fig. 4 for which the normalized intensity is represented as a function of detection angle with respect to Z/normal axis. From these panels, it is observed that there is good agreement between the results obtained by simulation and those measured experimentally. With the presence of the zero-index metamaterial at both sides of the aperture, first, it is possible to enhance light transmission from the aperture. And, second, the emitted power can be confined to a very narrow angular region with a half-power-width of almost 20° (from experiment) compared to the widespread emitted power of the bare aperture.

### Enhanced light transmission and beaming with an epsilon-near-zero material

**Figure 5:**
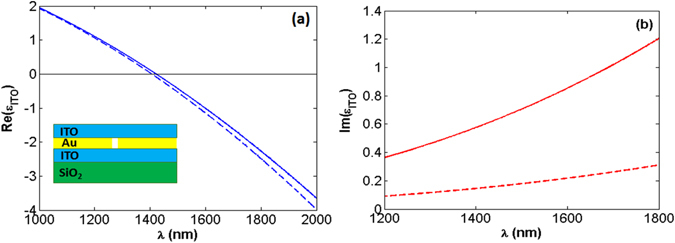
Solid curves in panels (a) and (b), respectively, illustrate the real and imaginary parts of epsilon of a realistic *ITO* with *γ*_*p*_ = 0.51 × 10^14^ *rad*/*s* ^30^. For the low-loss *ITO* case, for which *γ*_*p*_ = 2.05 × 10^14^ *rad*/*s*, the data are represented in dashed curves.

As a complementary of the results we obtained in the previous part, here we numerically investigate the effect of the presence of *ITO*, as an homogeneous epsilon-near-zero medium, on transmission of light through a subwavelength-width gold aperture. As schematically illustrated in the inset of Fig. 5(a), the aperture which is bounded by ITO is placed on a *SiO*_2_ substrate. For this structure, the width of the gold aperture, its thickness and the thickness of *ITO* layers are taken as 300 *nm*, 50 *nm*, and 80 *nm* in the calculations, respectively. Using the Drude dispersion *ε*(*ω*) = *ε*_∞_ − ω_*p*_^2^/[*ω*(*ω* + *iγ*_*p*_)], with *ε*_∞_ = 3.91, *ω*_*p*_ = 2.65 × 10^15^ *rad*/*s*, and *γ*_*p*_ = 2.05 × 10^14^ *rad*/*s*, the real and imaginary parts of the permittivity of a realistic (lossy) *ITO* ^32^ are represented in solid curves in Fig. 5(a) and (b), respectively. Moreover, the dashed curves in this figure belong to a low-loss *ITO* case for which *γ*_*p*_ = 0.51 × 10^14^ *rad*/*s* [*γ*_*p*_ (low-loss) = *γ*_*p*_ (lossy)/4]. It is noteworthy that, here, by the investigation of the effect of the presence of the low-loss/lossy *ITO* on transmission and beaming characteristics of the system, we will be able to understand how the loss reduction of the ENZ medium enhances the efficiency of the structure. From a practical point of view, for the *ITO*/*A*/*ITO* structure under our consideration in this section, it is not possible to fabricate the low-loss ITO with the loss factor 4 times smaller than that of a real ITO. Our purpose of investigating the results with the low-loss ITO is to prove this point that decreasing the losses in an ENZ medium leads to simultaneously achieving considerable enhanced transmission and beaming for the *ITO*/*A*/*ITO* system. This point also supports the results we obtained by the *ZIM* metamaterial. In other words, the investigated *ZIM* metamaterial in the previous section can be a practical alternative for the low-loss ITO.

**Figure 6:**
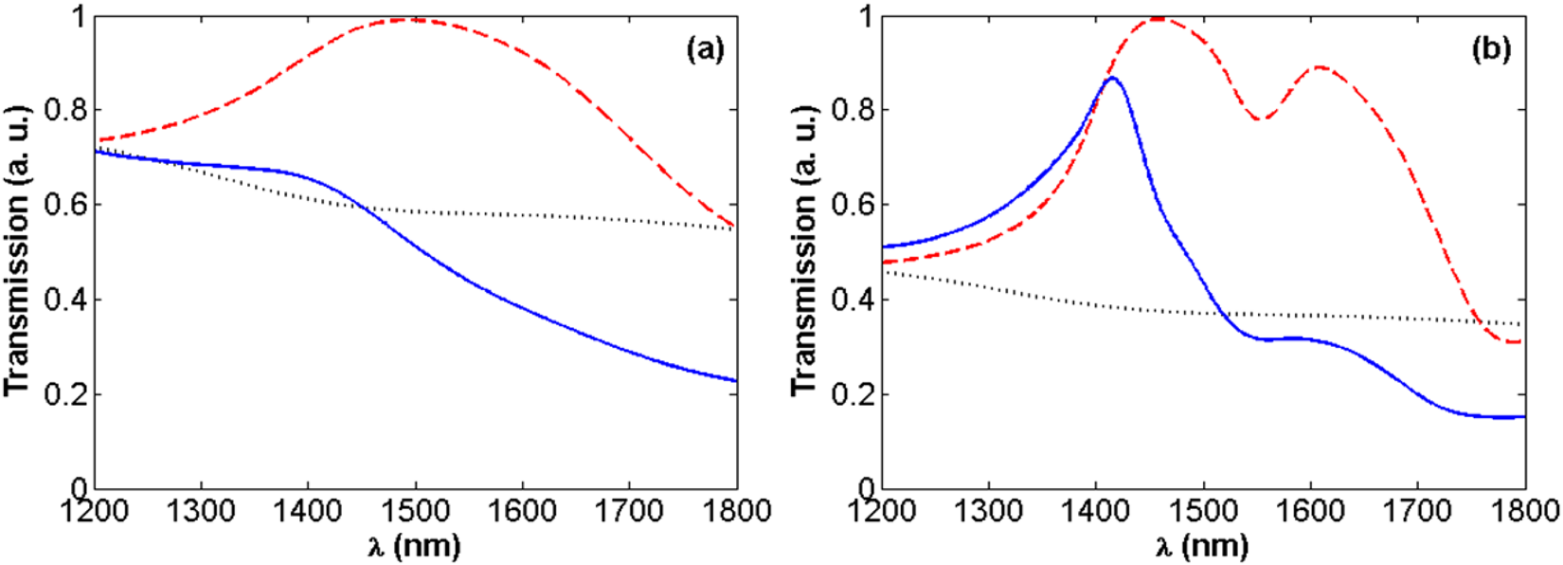
Transmission of light through the bare aperture (black-dotted), *ENZ*/*A* system (dashed-red), and *ENZ*/*A*/*ENZ* hybrid structure (solid-blue) considering lossy [panel (a)] and low-loss ITO [panel (b)] in the calculations, for Δ = 0. Δ is the dielectric spacer layer separating the aperture and the *ENZ* layers. Notice that the results are normalized to the maximum value (resonance peak) of the dashed-red curves.

As can be seen in panel (a) of Fig. 5, both realistic and low-loss *ITO* materials act as *ENZ* media for 1190 *nm* < *λ* < 1600 *nm*, in which |*Re*(*ε*)| < 1. Moreover, as it is illustrated in panel (b) of this figure, due to the a four-fold decrease in the collision rate of the carriers (*γ*_*p*_), a considerable reduction in the imaginary part of the permittivity of the *ITO* sample is observed. This reduction leads to a decrease in the losses of the *ENZ* medium, and thereby an increase in the transmission of the system. First, we investigate transmission of light through the subwavelength gold aperture which is bounded either by the realistic or low-loss *ENZ* materials on top of *SiO*_2_ as the substrate (see Fig. 6).

As presented in Fig. 6(a), when we place the realistic *ENZ* medium at the inner side of the aperture, light transmission is noticeably increased. In fact, as illustrated in Fig. S2(a) of the Supporting Information, in this case it is possible to achieve an almost 1.75 times enhancement in the transmission of light through the aperture. Moreover, while the aperture is covered on both sides by the realistic *ENZ* material, because of the lossy nature of *ITO*, light transmission is drastically dropped and for some wavelengths it is almost filtered. On the other hand, when we place low-loss *ITO* material as an *ENZ* medium at the inner side of the aperture, a higher increase in light transmission can be observed, as compared with the lossy case. This point can be understood by the comparison of the dashed-red curve in panel (b) of Fig. 6 with the one shown in panel (a) of this figure. In this case, it is possible to achieve a 2.7 times enhancement in light transmission, which is directly related to the decrease in the losses of the *ENZ* medium [see Fig. S2(b)]. Moreover, on the contrary with the lossy case, when the aperture is covered on both sides with the low-loss *ENZ* medium, as represented by the solid-blue curve in Fig. 6 (b), it is still possible to achieve a noticeable enhancement in light transmission in the *ENZ* region. The solid-blue curve in Fig. S2(b) shows that this enhancement is almost 2.4 times at 1415 *nm*. Furthermore, an additional resonance at 1600 *nm* is also observed in the dashed-red curve in Fig. 6 (b). This resonance which is placed at the edge of the *ENZ* region can be more pronounced by further decrease in the losses of the *ENZ* medium. Consequently, in case the losses are sufficiently decreased (e.g. *γ*_*p*_ = 2.92 × 10^13^ *rad*/*s*), it is even possible to obtain larger enhancement values of light transmission for the *ENZ*/*A*/*ENZ* system compared to that of the *ENZ*/*A* structure. Another important point that should be highlighted here is that, as illustrated in Fig. S3, by having a dielectric spacer of width Δ in-between *ITO* and the aperture, it is possible to red-shift the resonance wavelength within the *ENZ* region. In this way, by appropriate design of the hybrid structure, it is possible to observe the enhanced transmission *at a desired frequency* inside the *ENZ* region. As was already mentioned in the previous section, this tuning characteristic also works for the *ZIM*/*A*/*ZIM* metamaterial structure; i.e. by increasing Δ the enhancement frequency, which is placed inside the *ENZ* region for Δ~2 *mm*, can be red-shifted to the index-near-zero region.

**Figure 7:**
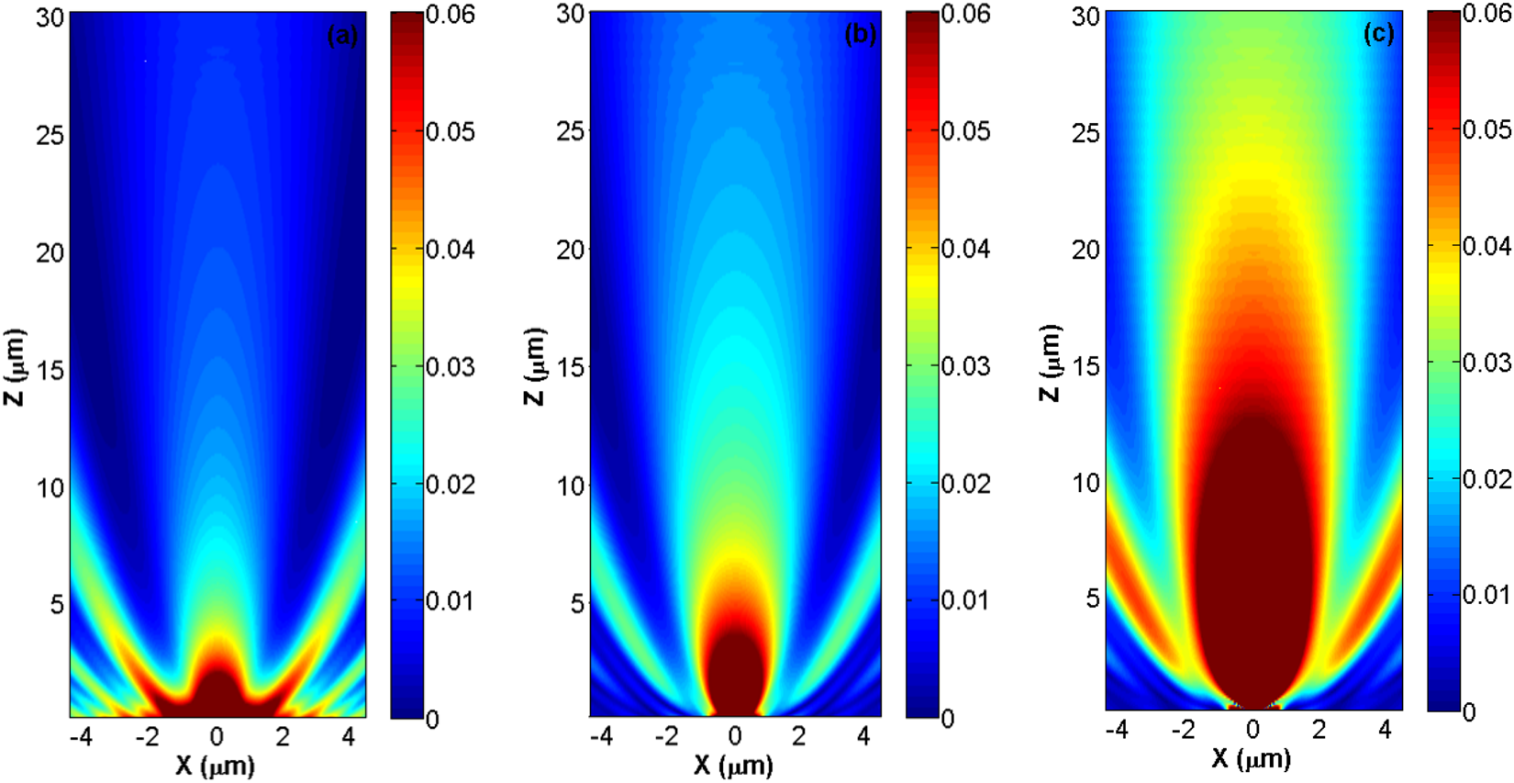
Numerical illustration of the far-field distribution of the electric field intensity of the extracted light from a bare aperture [panel (a)], the one that is covered by a realistic *ITO* [panel (b)] and low-loss *ITO* as *ENZ* media [panel (c)], for Δ = 0 at *λ* = 1415 *nm*.

Following the discussions presented in the previous section, we would like to investigate the beaming characteristic of the *ENZ*/*A*/*ENZ* hybrid structure. Figure 7 illustrates the far-filed distribution of the electric field intensity of light extracted from a bare aperture [Fig. 7(a)], an aperture covered by the realistic *ITO* [Fig. 7(b)], and the one which is bounded with the low-loss *ITO* as an epsilon-near-zero medium [Fig. 7(c)]. By comparing Fig. 7(a) and (b), it is seen that the presence of the lossy *ITO* at the both sides of the aperture, in addition to slightly enhancing light transmission, beams its extractions. However, as clearly observed from panel (c) of Fig. 7, by decreasing the losses of the *ENZ* medium, it is possible to considerably enhance light transmission in the *ENZ*/*A*/*ENZ* structure, as well as to noticeably beam its extraction. These results strongly support the ones concluded from panels (c)–(f) of Fig. 3, stating this point that using an appropriately designed low-loss *ENZ metamaterial*, it is possible to simultaneously achieve enhanced transmission and beaming of light using *ENZ*/*A*/*ENZ* hybrid structure.

**Figure 8:**
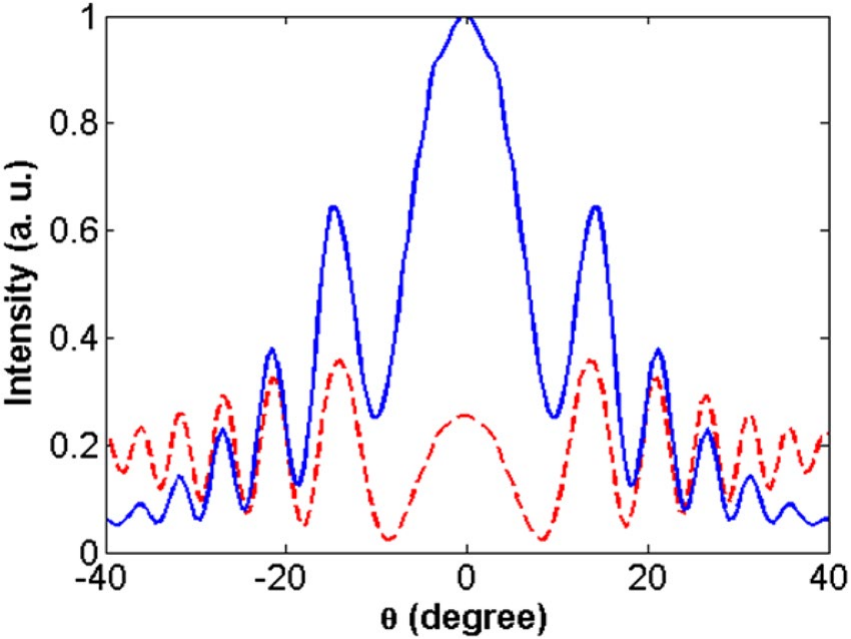
Far-field angular radiation patterns of a bare aperture (dashed-red) and an aperture which is covered with a low-loss ITO as an ENZ medium (solid blue) for Δ = 0 at *λ* = 1415 *nm*.

Furthermore, in agreement with Fig. 7(c), Fig. 8 shows the directionality of the low-loss *ENZ*/*A*/*ENZ* system. This figure completely meets the points understood from Fig. 4, supporting this idea that the presence of a low-loss *ENZ* metamaterial at the inner and outer sides of an aperture enhances light transmission and makes its extraction directional. As a results, the outcomes of this section fully reads the concluded remarks regarding the zero-index metamaterial that we discussed in the previous part.

## Conclusion

In conclusion, we have numerically and experimentally verified that the presence of an epsilon-near-zero metamaterial at the entrance side of a subwavelength aperture leads to an outstanding enhancement of light transmission through the aperture in the microwave region. This enhancement comes from the strong coupling between free space and the aperture due to the amplification of light passing through the metamaterial. Moreover, it has been found that the resonance frequency is tunable with the width of a spacer layer in between the aperture and the metamaterial. In other words, by appropriate width of the spacer layer, the resonance frequency of the system can be supported in either epsilon-near-zero or index-near-zero region. We have also demonstrated that in case the aperture is covered by the metamaterial on both sides, in addition to achieving a considerable enhancement of light transmission, it is also possible to intensely beam the extracted light. Moreover, as a proof of concept, we have numerically verified our results in mid-IR by the investigation of transmission and directionality of the light through a subwavelength gold aperture that is covered by *ITO* as an ENZ medium. The designs presented in this paper can be potentially employed in miniaturized devices that are beneficial for near-field microscopy, lasing, and light extraction from LEDs.

## Methods

The experimental set up that we used to measure light transmission consists of a network analyzer and two standard-gain horn antennas, which is similar to our previous studies^3, 30, 36^. Radiation is normally incident upon the system from 15 *cm* by the source antenna, and the receiver antenna is 100*cm* away from the structure back face. To measure the angular dependence of the far-field radiation, the receiver antenna was connected to a rotating arm. The schematic of the experimental setup is illustrated in Fig. 2 of ref. 36. Furthermore, we performed three-dimensional Finite Difference Time Domain (FDTD) calculations for the investigation of transmission spectra of the systems under our consideration^35^. In these calculations, the total field scattered field source was used to simulate the interaction between an incident plane wave and the hybrid structures. Furthermore, for the metallic parts of the considered systems we chose PEC and gold, for GHz and mid-IR calculations, respectively. It is noteworthy that the experimental data^37^ with a multi-coefficient fit in mid-IR region have been used for the optical constant of the gold aperture.

## Electronic supplementary material

Supplementary Information
