# Supplementary material for: Beaming and enhanced transmission through a subwavelength aperture via epsilon-near-zero media
Source: Sci Rep. 2017 Jul 6;7:4741. doi: 10.1038/s41598-017-04680-y (PMC5500505; doi:10.1038/s41598-017-04680-y)
Supplement: Supplementary file 1 — Supplementary Information [file 41598_2017_4680_MOESM1_ESM.pdf]

Beaming and enhanced transmission through a subwavelength aperture via epsilon-near-zero media

H. Hajian[1*], E. Ozbay[1,2,3,4], and H. Caglayan[1*]

[1]Nanotechnology Research Center, Bilkent University, 06800 Ankara, Turkey

[2]Department of Physics, Bilkent University, 06800 Ankara, Turkey

[3]Department of Electrical and Electronics Engineering, Bilkent University, 06800 Ankara, Turkey

[4]UNAM-Institute of Materials Science and Nanotechnology, Bilkent University, 06800 Ankara, Turkey

[*]corresponding authors: hodjat.hajian@bilkent.edu.tr andhcaglayan@bilkent.edu.tr

1. Enhancement of light transmission through the subwavelengthaperture

In this section, we investigate the enhancement of light transmission through the 3 mm sub-wavelength aperture. As shown in Fig. S1(a), the numerical results show that an eightfold enhancement can be obtained for the case wherein we have the *ZIM* (fishnet metamaterial) at the inner side of the aperture. In agreement with the numerical results, the dashed-red curve in panel (b) of this figure experimentally illustrates a sevenfold enhancement for the light transmission of the *ZIM/A* system. Moreover, our experimental results in Fig. S1(b) representa threefold enhancement for the case wherein we cover the aperture on both sides with *ZIM*. Moreover, it is observed that there is good agreement between the numerical results and the experimental ones in the observation of the resonance frequency at 12.7 GHz.

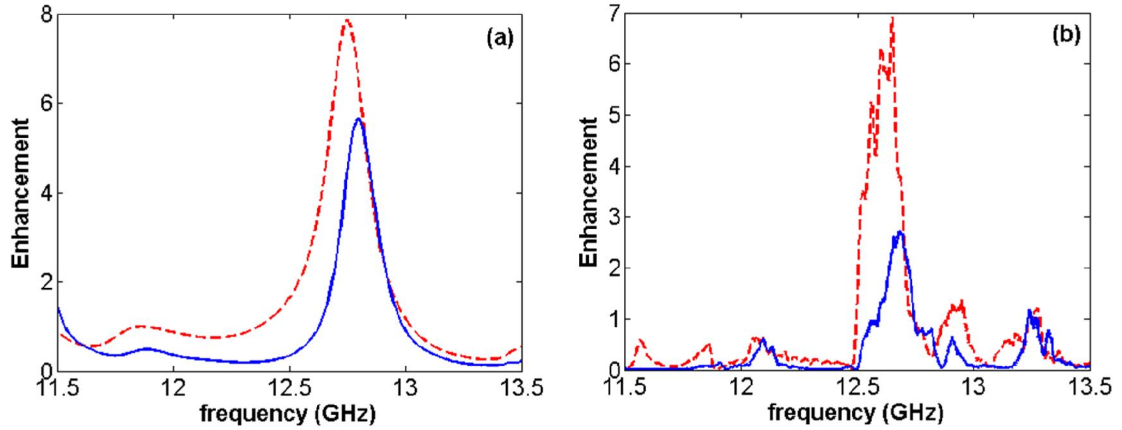

Supplementary Figure S1. Panels (a) and (b), respectively, illustrate the simulated and measured enhancement in thetransmission of light passing through the subwavelength aperture, as shown in Fig. 1(c), for *ZIM/A* (dashed-red curve) and *ZIM/A/ZIM* (blue curve) structures. These panels are represented in agreement with Figs. 3(a) and 3(b).

2. Enhancement of light transmission through the subwavelength gold aperture

In agreement with Figs. 7(a) and (b), enhancements of light transmission through a 300 nm gold aperture are numerically illustrated in Fig. S2. As is shown by the dashed-red curve in Fig. S2(a), wherein the ENZ medium (ITO) is placed at the inner side of the subwavelength aperture, 1.75 times the enhancement of light transmission can be observed. Moreover, when wecover the aperture with this material on both sides, because of the losses of the ENZ medium no enhancement in light transmission through the system can be obtained. On the other hand, when we decrease the losses, light transmission can be approximately enhanced 2.7 and 2.4 times for the *ENZ/A* and *ENZ/A/ENZ* cases, respectively (please see panel (b) of Fig. S2).

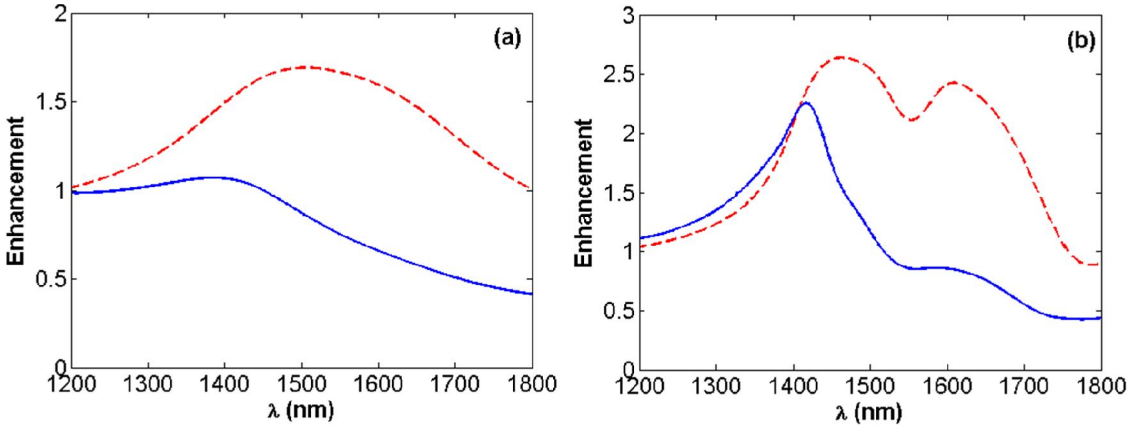

Supplementary Figure S2. In agreement with Figs. 7(a) and 7(b), the enhancement of light transmission through a 300 nm gold aperture considering ENZ and low-loss ENZ media is shown in panels (a) and (b), respectively. The dashed-red and solid-blue curves correspond to *ENZ/A* and *ENZ/A/ENZ* cases, respectively. Notice that Δ=0 in these results.

3. Tuning of the enhanced transmission of light through the subwavelength gold aperture

Another important point regarding our results is the tunable characteristics of the resonant modes versus the change in Δ. Similar to the schematics represented in Fig. 1(c), Δ is the thickness of the gap separating the subwavelength aperture and *ENZ* medium. For the numerical results shown in Fig. S3, this gap is filled with SiO_2. As is shown in Fig. S3(a), by increasing this gap to 100 nm, for the case of *ENZ* medium (ITO), it is possible to slightly shift

the resonance peak to larger wavelengths. Moreover, in case the losses of the epsilon-near-zero material are decreased, a noticeable red-shift of the resonance peak can be obtained in the *ENZ* region, for *ENZ/A* and *ENZ/A/ENZ* structures (please check dashed-red and solid-blue curves in panel (b) of Fig. S3, respectively).The ZIM structure that we experimentally investigated in the paper acts very similar to the low-loss ENZ medium discussed in Fig. S3(b); i.e. by decreasing the thickness of the air-gap in-between aperture and the metamaterial, it is possible to red-shift the enhancement/beaming frequency from 12.7 GHz. As a result, by doing so, it is possible to tune the resonance frequency.

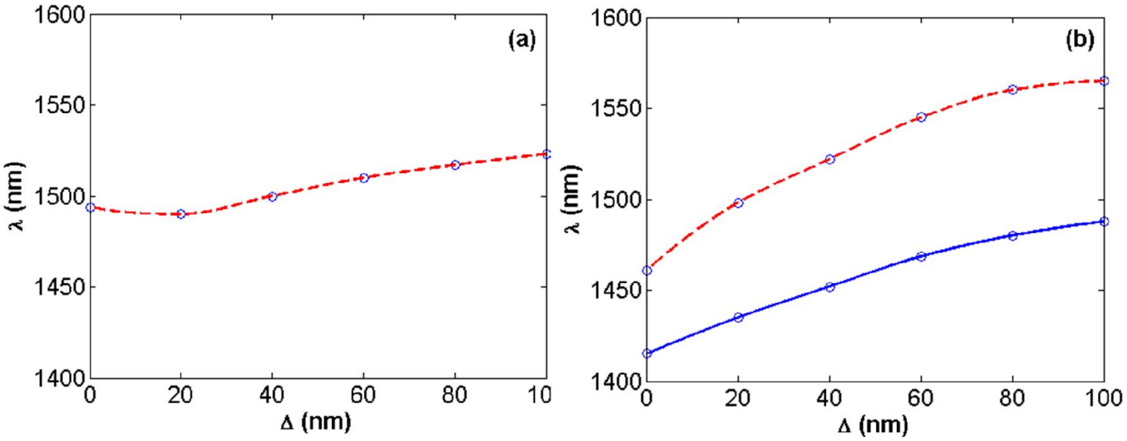

Supplementary Figure S3. The effect of changes in the thickness of an SiO_2 spacer layer (Δ) placed in-between the 300 nm gold aperture and the ENZ medium for real ITO [panel (a)] and low-loss ITO [panel (b)] cases. Dashed-red and solid-blue curves correspond to *ENZ/A* and *ENZ/A/ENZ* cases, respectively.
